# The moderating role of sportsmanship and violent attitudes on social and personal responsibility in adolescents. A clustering-classification approach

**DOI:** 10.1371/journal.pone.0211933

**Published:** 2019-02-07

**Authors:** Javier Courel-Ibáñez, Bernardino Javier Sánchez-Alcaraz, Alberto Gómez-Mármol, Alfonso Valero-Valenzuela, Juan Antonio Moreno-Murcia

**Affiliations:** 1 Department of Physical Activity and Sport, Faculty of Sport Sciences, University of Murcia, Spain; 2 Department of Plastic, Musical and Dynamic Expression, Faculty of Education, University of Murcia, Spain; 3 Department of Health Psychology, Faculty of Sport sciences, University Miguel Hernández of Elche, Alicante, Spain; TNO, NETHERLANDS

## Abstract

The aim of this study was to classify a randomized sample of adolescents according to their violent and sportsmanship attitudes to determine the influence of personal and social responsibility levels on each cluster. The sample comprised of 595 adolescents, aged between 12 and 15 years old (M ± SD = 13.9 ± 2.3 yr). Participants completed the Multidimensional Orientations Towards Sports Scale, the California School Climate and Safety Survey and the Personal and Social Responsibility Questionnaire. Cluster analysis was conducted to classify adolescent’ profiles according to the violence and sportsmanship scores. Discriminant analysis, Pearson correlation and ANOVA tests were performed to identify the relationships between personal and social responsibility levels on each cluster. Cluster analysis identified three well-defined profiles: cluster 1 (sportsmanlike and nonviolent), cluster 2 (sportsmanlike and violent) and cluster 3 (unsportsmanlike and nonviolent). Results confirmed a negative impact of aggressiveness on obedience and pro-social behaviours during school stages, but sportsmanship mitigated this negative influence. The sportsmanlike and nonviolent profile obtained the highest personal and social responsibility level. The lowest personal responsibility scores came from sportsmanlike and violent adolescents. This study emphasizes the potential of positive attitude towards sport and physical education to magnify (or mitigate) adolescents’ responsibility according to specific profiles. To reduce violent behaviour and improve sportsmanship attitudes would contribute to a better development of personal and social responsibility in adolescents. The current findings may serve to orientate professionals dealing with adolescents in the role of sportsmanship development as an educational tool to mitigate violent behaviour.

## Introduction

The school environment largely determines adults’ attitude to face life, chiefly during developmental stages like the adolescence [1]. Aggressiveness in schools has been associated with negative social and personal outcomes, health and mental risk factors, and early bad habits that stay in older age [2]. In light of this impact, there is a necessity to carry out school-based interventions as a pedagogical instrument to dismiss violence and encourage students’ integral development [3,4].

Evidence revealed positive effects of personal and social responsibility programs in decreasing scholar violence, particularly through physical education lessons, exercise activities and values in sport [5,6]. Specifically, increments in sportsmanship and fair play appear to be effective in preventing aggression in terms of active participation and passive encouragement [7,8]. In this context, some studies pointed out how personal and social responsibility development improves sportsmanship, self-control and fair play [9–11] and decreases aggressiveness [12] and both observed and suffered violence [13]. Sportsmanship is positively related to pro-social behaviour involving colleagues, and negatively to antisocial behaviour [5]. Responsibility relies on pro-social behaviour, empathy and self-efficacy behaviour, but is mitigated by aggressiveness [7]. Thus, education in personal and social responsibility appears to be an effective tool to decrease violent behaviour.

It is worth noting that adolescents are likely to change their attitudes and behaviours due to the media, the social pressure or friends influence, both in a positive and negative way [14,15]. The mere observation of violence on television can cause changes towards aggressive behaviour in the short and long terms [15]. Previous studies identified antisocial and violent behaviour in boys aged 11 to 13 years as a consequence of repeated contact with negative attitudes, norms, and ideals [16]. Furthermore, increments in unsportsmanlike play, cheating and aggressiveness could be expected when setting ego-oriented goals (i.e., emphasis on winning as opposed to developing skill) rather than task-oriented goals (i.e., evaluate success by effort and improvement) [17]. This hostility tends to increase with age among high school students [18]. Hence, teachers should be sensitive to these influences among students when designing cooperative or competitive activities to ensure compliance with the learning objectives and students’ commitment in a motivational environment [19,20].

A better knowledge of students’ sportsmanship and violent profiles (i.e., more or less violent, more or less sportsmanship) would orientate educators in designing teaching strategies in group activities [17,21]. To this task, classification methods in clusters appears to be a useful solution, as organized individuals in groups according to multiple factors [22]. This technic has been successfully used for educational purposes to determine motivational profiles [23] or identify academic goals [24] with substantial theoretical and practical value. A recent review [25] remarked on the need for further research on the role of personality as a protective factor of aggression in school, and the development of strategies to promote and strength preventive behaviours through sport. However, to the best of our knowledge, there is still no study examining adolescents’ profiles according to personal and sportsmanship behaviours. Thus, there is no data regarding the potential protective effect of developing responsibility and sportsmanship attitudes in reducing violence in school. Given the above-mentioned issues, this information would provide educators with critical tools to improve school interventions plans and create school environments to reduce antisocial and violent behaviours in youth.

Hence, in this study we aimed to classify a randomized sample of adolescents according to their violent and sportsmanship attitudes, and to determine relationships on their personal and social responsibility levels. It was hypothesised that positive attitude towards sport and physical education could mitigate aggressiveness and increase adolescents’ responsibility. In addition, we expected different responses according to specific profiles.

## Methods

### Participants

This cross-sectional study involved 595 adolescents (262 girls), aged between 12 and 15 years old (M ± SD = 13.9 ± 2.3 yr). Sixteen schools from Murcia (Spain) were randomly selected according to the following inclusion criteria: one primary school and one secondary school from each of the eight representative areas from the Region of Murcia, according to the territorial division provided by the Teachers and Resources Centres. The sample was recruited from eight primary and eight secondary schools. Two classes of around 20 adolescents each were randomly selected per school.

### Instruments

#### Sportsmanship

The Multidimensional Sportspersonship Orientations Scale [26] was used to measure the level of sportsmanship. This scale consists of 25 items structured into 5 dimensions: (i) personal commitment to doing sport, (ii) social conventions, (iii) respect for rules, judges and referees, (iv) respect towards opponents and (v) negative perspective. To evaluate the dimensions, participants were asked to answer the question: “which of the following expressions do you consider form part of sportsmanship?” (see Table 1 a summary of the instrument). The responses are given on a Likert scale, from (1) *totally disagree* to (5) *totally agree*. The internal consistency obtained was the following: commitment to doing sport (*α* = .61), social conventions (*α* = .78), respect for rules, judges and referees (*α* = .67), respect towards opponents (*α* = .62), and negative perspective (*α* = .68). Given the reduced number of items for each factor, the obtained internal consistency was considered acceptable [27].

**Table 1:** Summary of instruments used to evaluate adolescents.

Questionnaire / Dimension	Questions
**Sportsmanship**	Main question: *which of the following expressions do you consider form part of sportsmanship*? [1–5 Likert response to the following:]
Commitment to doing sport	E.g. “*I try to get involved in all activities*”.
Social conventions	E.g. “*to congratulate your opponent for having played well*”
Respect towards rules and judges	E.g. “*to respect the referee even if he makes a mistake*”
Respect towards opponent	E.g. “*to rectify a situation which is unfair for the opponent*”
Negative sportsmanship	E.g. “*to make excuses for playing badly*”
**Violence**	Main question: *Answer whether the following has taken place in your classroom this year* [1–5 Likert response to the following:]
Violence experienced	E.g. “*I was punched or kicked*”.
Violence observed	E.g. “*Students get into fights*”.
**Social and Personal responsibility**	Main question: *The normal thing is to sometimes behave well and other times badly*, *we would like to know how you normally behave during the physical education class* [1–6 Likert response to the following:]
Social responsibility	E.g. “*I respect others*”.
Personal responsibility	E.g. “*I want to improve*”.

#### School violence

The modified version of the California School Climate and Safety Survey [28,29] includes 14 items, conceptually adequate, to evaluate school violence among peers. The items are classified into two dimensions: violence experienced and violence observed. Each item was preceded by the sentence: “Answer whether the following has taken place in your classroom this year” (see Table 1 a summary of the instrument). Participants were asked to give a value on a Likert scale, which ranges from (1) *never* to (5) *always*. The internal consistency obtained was the following: violence experienced (*α* = .84) and violence observed (*α* = .84).

#### Personal and social responsibility

The Personal and Social Responsibility Questionnaire [30] was used to measure participants’ personal and social responsibility. The questionnaire consists of 14 items, distributed into two factors–seven for each one: personal responsibility and social responsibility, preceded by the sentence: “The normal thing is to sometimes behave well and other times badly, we would like to know how you normally behave during the physical education class” (see Table 1 a summary of the instrument). The participants must respond on a 6-point Likert scale, which goes from (1) *totally disagree* to (6) *totally agree*. The internal consistency obtained was the following: personal responsibility (*α* = .67) and social responsibility (*α* = .82).

### Procedures

Students completed the three questionnaires voluntarily and anonymously in the same day, during school hours. The same researcher was present in the classroom during survey delivery in all centres. Adolescents answered the questionnaires in about 40 minutes (timeline was 60 minutes) and none of students reported any problems completing them. The study was approved by the Ethical Committee of the University of Murcia (R-593/2009). Written informed consent was obtained from all participants and adolescents’ parents.

### Statistical analysis

All variables are provided as means ± standard deviations (SD). Records were screened for univariate outliers (cases outside the range Mean ± 3SD). A hierarchical cluster analysis using Ward’s method was performed to create several groupings according to the results of sportsmanship and violence tests [31]. Squared Euclidean distance between observations was computed as the dissimilarity measure. The *NbClust R* package was used to determine the optimal number of clusters from the dataset [32]. The Pearson correlation coefficient was used to measure associations between sportsmanship and violence within each group. Afterwards, a descriptive discriminant analysis was performed to identify which of the variables best predicted the behavioural clusters. Validation of discriminant models was conducted using the leave-one-out method of cross-validation [33]. Finally, factorial ANOVA and post-hoc pairwise comparisons were used to identify differences in personal and social responsibility punctuations between the sportsmanship and violence clusters solutions. Significant interactions were further investigated using unpaired t-tests with Bonferroni correction, i.e., the level of significance for individual comparisons was set at *p* < 0.016 to ensure a global type I error rate of 0.05 [34]. Between-group effect sizes (ES) were calculated using Cohen’s *d* (distance in number of standard deviation units between clusters), Cohen’s U3 (the percentage of one cluster which the upper half of the cases of the other cluster exceeds) and the probability of superiority CL (the probability that a person picked at random from one cluster will have a higher score than a person picked at random from the other cluster) coefficients [35,36]. Cohen’s *d* was interpreted as: trivial = 0–0.19; small = 0.20–0.49; medium = 0.50–0.79; and large = ≥ 0.80. Calculations were performed using R version 3.2.1 (R Foundation for Statistical Computing, Vienna, Austria) and SPSS version 22.0 (IBM Corp., Armonk, NY, USA) for windows.

## Results

According to the majority rule from *NbClust* (12/30 indexes), the best number of clusters was three (n(cluster1) = 271; n(cluster2) = 186; n(cluster3) = 138). Table 2 presents means, standard deviations and structure coefficient of variables according to the cluster solutions. The discriminant analysis revealed two statistically significant functions (F = 7.639; *p* ≤ 0.001) with canonical correlations of 0.785 (57.2% of variance) and 0.739 (42.8% of variance), respectively. The reclassification of the cases in the original groups was very high (96.3%). Function 1 was characterized by commitment to doing sport (*r* = -.649), social conventions (*r* = -.279), respect towards rules and judges (*r* = -.251), and respect towards opponent (*r* = .174). Function 2 was however emphasized for negative sportsmanship (*r* = -.692), violence experienced (*r* = -.386) and violence observed (*r* = .272).

**Table 2:** Means, standard deviations and structure coefficient of variables according to the cluster solutions.

Cluster / Variable	Cluster 1	Cluster 2	Cluster 3	Function 1	Function 2
**Sportsmanship**					
Commitment to doing sport	4.55 ± 0.44	4.34 ± 0.55	3.69 ± 0.82	.108*	.091
Social conventions	4.67 ± 0.41	4.21 ± 0.78	3.30 ± 0.90	.064*	.230
Respect towards rules and judges	4.62 ± 0.43	4.41 ± 0.46	3.35 ± 0.87	.228*	.160
Respect towards opponent	3.71 ± 0.79	2.63 ± 0.76	2.78 ± 0.81	.618	.638*
Negative sportsmanship	4.31 ± 0.58	3.93 ± 0.76	3.22 ± 0.74	.387*	-.320
**Violence**					
Violence experienced	1.26 ± 0.30	1.82 ± 0.78	1.63 ± 0.63	-.123	.303*
Violence observed	1.69 ± 0.63	3.01 ± 0.72	2.12 ± 0.84	-.707*	.505

*Higher combined intra-group correlation.

**Fig 1:**
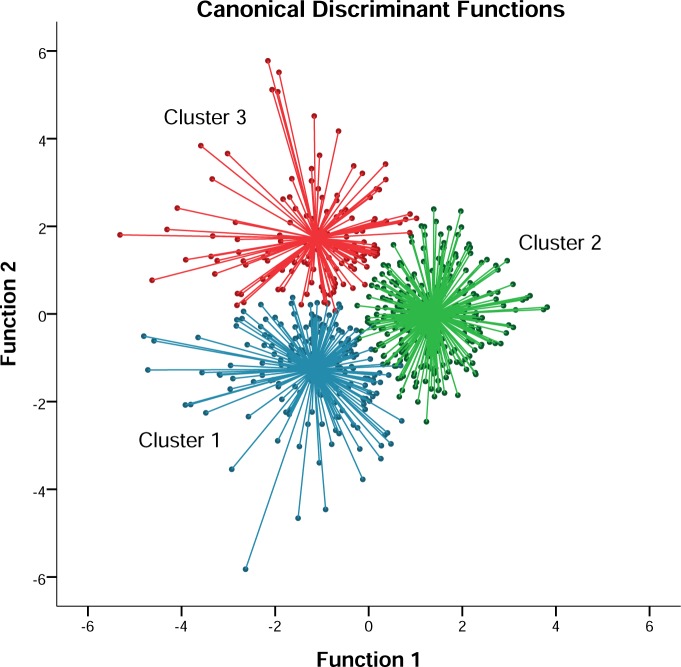
Distribution of adolescents in clusters according to canonical discriminant functions.

Discriminant analysis confirmed the stability of the grouping classification, showing a probability over 91.3% of belonging to the given cluster. Fig 1 depicts the territorial map from the cases according to the canonical discriminant functions. Cluster 2 was mainly represented by the Function 1, whilst Cluster 1 and 2 were positive and negative respectively characterized by the Function 2.

**Fig 2:**
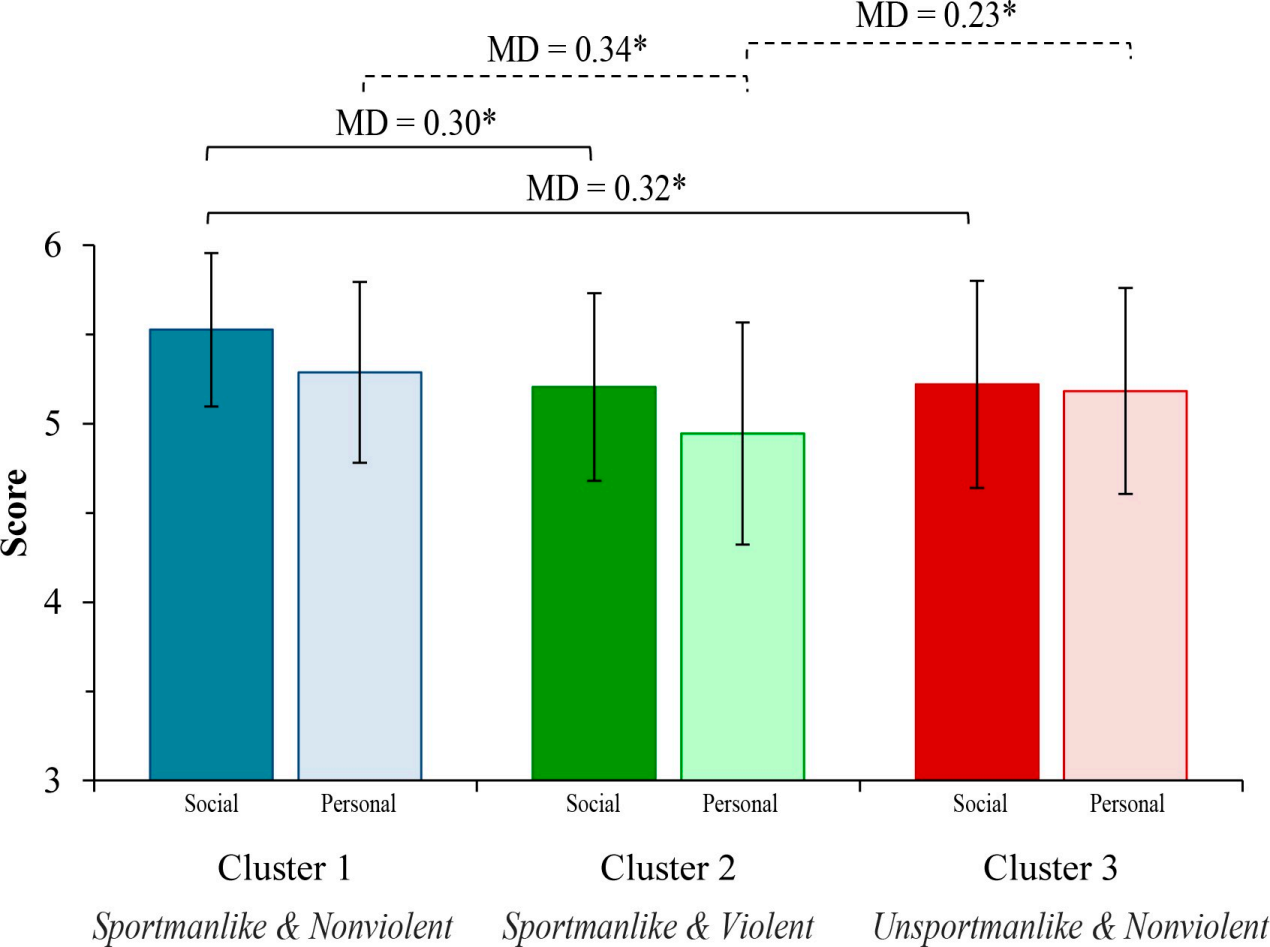
Social (dark bars) and personal (light bars) responsibility mean differences between clusters. Data are Means with Standard Deviation (M±SD).

Finally, ANOVA revealed differences in social (F(3,494) = 29.380; *p* < 0.001) and personal (F(3,494) = 20.822; *p* < 0.001) responsibility between clusters. Pairwise comparisons are presented in Table 3 and depicted in Fig 2. Cluster 1 obtained a higher social (*p* < 0.001) and personal responsibility (*p* < 0.001) compared with Cluster 2. Similarly, Cluster 3 obtained a lower social (*p* < 0.001) compared with Cluster 1, but higher personal responsibility (*p* < 0.001) compared with Cluster 2.

**Table 3:** Mean comparisons in social and personal responsibility between clusters.

Cluster comparisons	Social responsibility				Personal responsibility		
	Mean (95%CI)	*d*	U3 (CL)		Mean (95%CI)	*d*	U3 (CL)
**Cluster 1 vs Cluster 2**	0.30* (0.18,0.43)	0.62	73.2 (67.0)	** **	0.10 (-0.03, 0.24)	0.20	57.9 (55.6)
**Cluster 1 vs Cluster 3**	0.32* (0.43,0.20)	0.68	75.2 (68.5)	** **	0.34* (0.47,0.21)	0.62	73.2 (67.0)
**Cluster 3 vs Cluster 2**	0.01 (0.14,0.12)	0.03	-51.2 (-50.8)		0.23* (0.38, 0.08)	0.40	65.5 (61.1)

CI = Confidence Interval.

*Significant differences (Student’s t-test *p*<0.017). Effect sizes: *d* = distance in number of standard deviation units between clusters; U3 = percentage of the first cluster which the upper half of the cases of the second cluster exceeds; CL = probability that a person picked at random from the first cluster will have a higher score than a person picked at random from the second cluster.

## Discussion

This study aimed to classify a sample of adolescents according to their violent and sportsmanship attitudes, and to determine relationships on their personal and social responsibility levels. Cluster analysis identified three well-defined profiles: cluster 1 (sportsmanlike and nonviolent), cluster 2 (sportsmanlike and violent) and cluster 3 (unsportsmanlike and nonviolent). Consistent with previous research [5,7,37], our study confirmed that a negative impact of aggressiveness on obedience and pro-social behaviours during school stages, but sportsmanship mitigated this negative influence in school stages. To the best of our knowledge, this is the first time exploring adolescents’ profiles according to sportsmanship and aggressiveness attitudes altogether. Additionally, we have detected relationships between adolescents’ profiles and their responsibility. The sportsmanlike and nonviolent profile obtained the highest personal and social responsibility level, while the lowest personal responsibility scores came from sportsmanlike and violent adolescents. Thus, our study emphasizes the potential of positive attitude towards sport and physical education to magnify (or mitigate) adolescents’ responsibility according to specific profiles. This information may serve as to orientate professionals dealing with adolescents in the role of sportsmanship development as an educational tool to reduce violent behaviour.

To the best of our knowledge, this is the first time classifying adolescents’ profiles according to their sportsmanship and violent attitudes has been done. Our data identified three well-differentiated groups of students. As expected, the profile with the highest sportsmanship also was the most calm. More interestingly, the most violent group showed the lowest respect towards opponents, however, reported great sport commitment and respect towards rules and judges. This positive attitude towards sport and rules reinforces the notion that sport practice in physical education lessons could be an attractive context to reduce school violence and aggression and promote respect between students [38]. Finally, a third profile reflected an apparent disinclination to doing sport which resulted in higher violent attitudes. This cluster classification confirms the relationship existing between the psychological variables analysed in adolescents [39,40]. A negative relation among violence and sportsmanship could be explain because adolescents with unsportsmanlike behaviours have a higher risk of expressing aggressiveness due to a low trait anger control and self-control [41,42]. However, certain emotional and personality aspects such as emotional instability, intolerance, lack of social skills, hostility and lack of confidence could increase the incidence of aggressive and unsportsmanlike behaviours [37]. On the other hand, greater sportsmanship is shown to be related with personal and social responsibility values such as effort, cooperation, respect, problem solving and conflict management [43].

Besides, previous research has identified similar behavioural differences in adolescents; however, behaviours can be vulnerable to contagion processes [44]. This is particularly relevant given that adolescents are strongly affected by or exert influence on colleagues and friends’ behaviour [14]. Therefore, it suggests developing school-based cooperative strategies–with constant teacher supervision—in which students interact between each other to deal with confrontations during practice and leading to positive behavioural engagement. To this purpose, physical education stands as a main educational tool to develop sport commitment values and reduce school violence and aggressions [38]. In line with our results, it might be helpful to identify and organize students according to their sportsmanship and violent attitudes to avoid a negative impact in practice within groups.

A main issue addressed in this study was the relationships between above-mentioned sportsmanship and violence profiles and their responsibility. This leads to the suggestion that sports and physical activities could reinforce obedience and pro-social behaviours in school stages [5,6]. Hence, knowledge of particular sportsmanship attitudes which may predict personal and social responsibility should be considered as a preliminary step before designing school-based interventions programmes during physical education lessons [2,45]. Information of this nature will let educators adjust teaching strategies according to the students’ characteristics and needs. Consequently, the intervention will better contribute to the developing students’ motivation and satisfaction [21]. For instance, it is more likely that a more aggressive attitude will be shown when the goal competition is oriented towards a result rather than towards learning or performance [17]. In this situation, the student suffers greater tension which would increase aggressive and abusive behaviours. According to our result, this negative impact will be even worse in unsportsmanlike and violent students, whose will try to make the best to compete and get the victory even by disrespecting their colleagues. In such kind of cases will required the inclusion of self-control strategies to mitigate violent reactions keeping students’ attachment to sport in a responsible and cooperative way. Subsequently, unsportsmanlike and nonviolent group should be carefully controlled in order to preserve non-violent behaviours but enhancing commitment to doing sports through encouraging their intrinsic motivation towards the practice [19]. In this sense, among other proposals, the Personal and Social Responsibility Model (TPSR) became one of the most commonly program using school-based physical-sports activity as a pedagogical instrument for encouraging students’ integral development [3,4]. Interventions using this model reported benefits in personal and social responsibility aspects and sportsmanship behaviours [30,46], and decrements in aggressiveness and violence [8,47].

In conclusion, reductions in violent behaviours and improve sportsmanship attitudes will significantly contribute to the better development of personal and social responsibility in adolescents. We were able to identify three different groups of students, which would require particular interventions according to their characteristics (e.g., self-control to mitigate violence, intrinsic motivation to encourage sport commitment). This study has some limitations that should be noted. Data was collected through questionnaires. These methods allowed us to evaluate around six hundred adolescents; however, it would be interesting for future research to apply a more objective method such as behavioural observations in the school context to replicate these results. We must recognize a limited internal consistency observed in the “commitment to doing sport” and “respect towards opponents” constructs. Besides, we were not able to provide sociodemographic variables (e.g. gender, education level or school context) which could influence the current student’s profiles. Finally, life experiences vary considerably during developmental stages, thus students’ profiles must be interpreted with caution. To date, there are no published studies classifying violence and sportsmanship profiles in adolescents. Thus, further studies are required to corroborate the current profiles. Likewise, future research should take into account other types of variables which could be related to personal and social responsibility, such as self-control, autonomy, leadership, motivation, pro-social behaviour, empathy and self-efficacy.

## Supporting information

S1 Dataset Database sportmanship and violence cluster. (SAV)
